# Polychlorinated biphenyls and risk of hepatocellular carcinoma in the population living in a highly polluted area in Italy

**DOI:** 10.1038/s41598-021-82657-8

**Published:** 2021-02-04

**Authors:** Francesco Donato, Marco Moneda, Nazario Portolani, Angelo Rossini, Sarah Molfino, Silvia Ministrini, Giovanni Battista Contessi, Silvia Pesenti, Giuseppe De Palma, Alice Gaia, Elena Zanardini, Claudio Vito Sileo, Michele Magoni

**Affiliations:** 1 Unit of Hygiene, Epidemiology and Public Health, University of Brescia, Viale Europa 11, 25123 Brescia, Italy; 2 Post-Graduate School of Public Health, University of Brescia, Brescia, Italy; 3 Department of Clinical and Experimental Sciences, Surgical Clinic, University of Brescia, Brescia, Italy; 4 Hepatology Unit, Spedali Civili General Hospital, ASST (Territorial Socio-Sanitary Agency) Spedali Civili Di Brescia, Brescia, Italy; 5 Unit of Occupational Health and Industrial Hygiene, University of Brescia, Brescia, Italy; 6 Unit of Occupational Health, Hygiene, Toxicology and Prevention, ASST (Territorial Socio-Sanitary Agency) Spedali Civili Di Brescia, Brescia, Italy; 7 ATS Brescia (Brescia Health Protection Agency), Brescia, Italy

**Keywords:** Hepatology, Cancer, Epidemiology, Translational research, Environmental sciences

## Abstract

Polychlorinated biphenyls (PCBs) are human carcinogens, based on sufficient evidence for melanoma and limited evidence for non-Hodgkin lymphoma and breast cancer. Few data are available for liver cancer, although PCBs cause it in rats and determined liver damage in poisoned people. We investigated the association between PCB serum levels and hepatocellular carcinoma (HCC) with a case–control study in a PCB-polluted area in North Italy. We enrolled prospectively 102 HCC incident cases and 102 age and gender-matched hospital controls. Serum concentrations of 33 PCB congeners were determined by a gas chromatograph coupled to mass spectrometry. Of 102 HCC cases, 62 who had lost < 3 kg of body weight in past 3 years were included in the analysis (67.7% males, mean age 68 years). The odds ratio (OR) for HCC for 3rd compared to 1st tertile of PCB distribution was 1.76 (95% confidence interval 0.62–5.03) for total PCB, adjusting for socio-demographic variables and risk factors for HCC by logistic regression. For most PCB congeners, ORs > 1.5 or 2 were found, although the 95% CIs included the null value for almost all of them. This preliminary study suggests that PCBs might play a role in HCC development.

## Introduction

Polychlorinated biphenyls (PCBs) are a group of organochlorine compounds industrially produced until the 1980s, and banned afterwards in most countries due to their environmental persistence and toxicity^1^. PCBs are included among the persistent organic pollutants (POPs) and accumulate in soil, plants and animals and along the food chain^1^. Experimental studies on animals and epidemiological studies on humans have demonstrated an association between PCBs and metabolic and endocrine diseases, damage to nervous systems and development and immunological and reproductive disorders^2^. Moreover, PCBs have been classified as “carcinogens to humans” (group I) by the International Agency for Research on Cancer, based on sufficient evidence of their carcinogenicity for melanoma and limited evidence for non-Hodgkin lymphoma and breast cancer^2^. For other sites and types of cancer, only sparse data were available.

The liver is a target organ of PCBs: animals exposed to PCBs show biochemical changes indicative of hepatocellular damage and liver dysfunction (altered levels of lipids) and of fat liver deposition^1^. Hepatotoxic effects were found also in humans with extremely high PCB intake in the intoxication incidents of Yusho and Yucheng^1^. An increased mortality from chronic liver disease and cirrhosis was observed in the Yucheng men, though not in women, in the early period after exposure^3^. Recently, an association between steatohepatitis and serum levels of various PCB congeners was found in a cross-sectional study on a residential US cohort with elevated PCB exposure^4^, and an association between PCB serum levels and unexplained ALT elevation was observed in a multicentre US cross-sectional survey^5^. PCB exposure causes liver cancer in rats, but occupational cohort studies showed inconsistent results as regards liver cancer mortality^6^, and no epidemiological study on the association between PCBs and HCC using biological measures of PCB exposure has been conducted so far, to our knowledge.

A chemical factory located in Brescia, the main town in the province, produced PCBs and other organochlorines from the 1930s to 1984 determining a heavy contamination of the soil, surface water sediments and locally produced food, including animal products (milk and cheese, meat, chicken and eggs)^7–9^. A study on a random sample of adults living in the town showed higher PCB serum concentrations than those usually found in people living in industrial area and not occupationally exposed, up to about 40,000 ng/g of lipid-adjusted serum total PCBs^10^.

Hepatocellular carcinoma (HCC) is the most common type of primary liver cancer, accounting for more than 80% of primary liver cancers worldwide. A high incidence of HCC has been registered in the Brescia area as compared to others in Italy^11,12^. A case–control study carried out in 1995–2001 on 464 cases and 824 controls showed that hepatitis C virus (HCV), hepatitis B virus (HBV) and consumption of more than 60 g of ethanol per day for at least one decade were responsible for about 90% of the total cases occurring in the area^13,14^, in agreement with findings from other countries^15^. Also a role of nonalcoholic fatty liver disease (NAFLD) and nonalcoholic steatohepatitis (NASH) in HCC development has been demonstrated recently^16,17^, but this condition seems still uncommon in our country, being responsible for about 5% of the total HCC cases in a recent large multicentre Italian study^18^. However, a role of other factors, including environmental toxics for HCC development, cannot be excluded. We carried a preliminary case–control study aimed to investigate the possible association between PCB exposure and HCC, taking account of the major risk factors for HCC in Western countries.

## Results

A total of 102 cases and 102 controls were enrolled. Among HCC cases, 40 said they had lost 3 kg and over of their body weight in the last 3 years. A comparison between HCC cases with and without recent weight loss showed that the former had significantly higher PCB serum levels than the latter: the medians of total serum PCBs were 1183 and 757 ng/g lipid, respectively (p = 0.02 by Mann–Whitney test). Among the 40 HCC cases with recent weight loss, no relationship was found between either quantity of, or time at, weight loss and PCB serum levels. Since HCC cases with recent weight loss may have higher PCB serum levels due to their release from lipid deposits into the blood, they were excluded from the analysis, which was therefore restricted to 62 HCC cases.

Gender, age, residence and education and main risk factors for HCC in both cases and controls are shown in Table 1.

**Table 1:** Socio-demographic variables and risk factors for hepatocellular carcinoma (HCC) in cases and controls.

	Cases (N = 62)	Controls (N = 102)
**Gender**		
Male	42 (67.7%)	69 (67.6%)
Female	20 (32.3%)	33 (32.4%)
**Age, years**		
Mean (range)	70.1 (50–84.4)	70.0 (47.3–85.8)
**Education, school years**		
≤ 8 years	49 (79.0%)	65 (63.4%)
> 8 years	13 (21.0%)	37 (36.3%)
**Residence**		
Main town	11 (17.7%)	36 (35.3%)
Province	51 (82.3%)	66 (64.7%)
**Risk factors for HCC**^**†**^		
HCV^‡^	26 (41.9%)	3 (2.9%)
HBV^§^	4 (6.5%)	2 (2.0%)
Heavy alcohol intake (60 + g/day)	13 (21.0%)	5 (4.9%)
None of them	19 (30.6%)	92 (90.2%)

^†^p < 0.01.

^‡^HCV infection alone + HCV and HBV co-infection + HCV infection and heavy alcohol intake (60 + g/day).

^§^HBV infection alone + HBV infection and heavy alcohol intake (60 + g/day).

Age and gender distribution were almost identical between cases and controls, whereas some differences were observed according to residence, with 17.7% of cases and 35.3% of controls living in the town, and education, with 21.0% of cases and 36.3% of controls with more than 8 school years. Of the HCC cases, 41.9% had HCV infection, with or without other risk factors, 6.5% had HBV infection, with or without a history of heavy alcohol intake, 21% had a history of heavy alcohol intake alone, and 30.6% had no risk factors. As expected, few controls showed one or more of the main risk factors for liver disease: 4.9% had HCV or HBV infection and 4.9% had a history of heavy alcohol intake. A total of 44 cases (71%) had a clinical history of chronic liver disease with or without evidence of cirrhosis.

The median, 90th percentile and range of lipid-adjusted serum concentration of all the PCB congeners and of the total PCBs in HCC cases and controls are shown in Table 2.

**Table 2:** Median, 90th percentile and range of lipid-adjusted serum levels of polychlorinated biphenyl (PCB) congeners and total PCBs, in ng/g lipids, in HCC cases and healthy controls.

IUPAC PCB congeners	Cases (N = 62)			Controls (N = 102)		
	Median	90th percentile	Range (min–max)	Median	90th percentile	Range (min–max)
PCB 28	0	0	0–0	0	0	0–124
PCB 74	0	26	0–37	0	19	0–82
PCB 99	0	25	0–49	0	19	0–100
PCB 105	0	0	0–0	0	0	0–57
PCB 118	20.5	70	0–128	14.5	45	0–241
PCB 138	92.5	232	0–349	86	173	14–511
PCB 146	4	24	0–35	9	20	0–41
PCB 153	178.5	412	35–699	159.9	327	23–691
PCB 156	21.5	42	0–63	19.5	33	0–74
PCB 157	0	0	0–17	0	0	0–15
PCB 167	0	12	0–27	0	12	0–33
PCB 170	62	132	0–196	61	96	0–209
PCB 172	0	15	0–26	0	12	0–20
PCB 177	0	0	0–15	0	0	0–21
PCB 180	234	516	52–779	214.5	352	34–810
PCB 183	9.5	31	0–57	9	20	0–44
PCB 187	29.5	64	0–127	26.5	49	0–138
PCB 189	0	0	0–0	0	0	0–11
PCB 194	56	132	0–266	49	90	0–231
PCB 196 + 203	30	61	0–108	28.5	50	0–88
PCB 201	25.5	53	0–133	22	40	0–96
PCB 206	0	0	0–0	0	0	0–24
PCB 209	0	19	0–53	0	15	0–195
Total PCBs	757	1787	95–2703	737.5	1365	92–3089

Of the 12 dioxin-like PCBs, only PCB 118 and PCB 156 were found, both at low levels. The PCB congeners found at the highest concentration in our study have endocrine or immunotoxic activities or can induce Phenobarbital (congeners: 118, 138, 153, 156, 170, 180, 194). Slightly higher values of the median and 90th percentile were found in HCC cases than controls for all the congeners and total PCBs, although the differences were not statistically significant using the non-parametric Mann–Whitney test. A comparison between 44 HCC patients who had unifocal nodule and 18 cases who had multifocal or diffuse cancer was also performed, which showed no statistically significant differences between them for all congeners and total PCBs (Suppl. Table 1). Accordingly, the comparison between early HCC cases and controls did not show statistically significant differences between them.

No difference was found in total PCB serum levels in HCC cases according to the presence or absence of at least one of the main risk factors for HCC (Table 3).

**Table 3:** Lipid-adjusted serum levels of total polychlorinated biphenyls (PCBs) in hepatocellular carcinoma (HCC) cases.

Risk factors for HCC	Total PCB serum levels (ng/g lipid)		
	No. (%)	Median (range)	90th percentile
HCV infection	23	745 (95–2029)	1560
HBV infection	3	949 (232–1161)	1161
Heavy alcohol intake	13	552 (208–2703)	1787
HCV + heavy alcohol intake	2	604.5 (273–936)	936
HBV + heavy alcohol intake	1	2613 (2613 -2613)	2613
HCV + HBV + heavy alcohol intake	1	145 (145–145)	145
At least one risk factor	43	745 (95–2703)	1787
No risk factor	19	909 (305–2596)	2407

The 43 HCC cases with one or more risk factors had slightly lower values of total PCBs than the cases without risk factors (medians of 745 and 909, respectively), although the difference was not statistically significant. No difference was found in PCB serum concentration according to residence in the main town vs rest of province, in both cases and controls, when adjusting for age.

The distribution of HCC cases and controls according to tertiles of PCB concentration in the controls, and the corresponding odds ratios (ORs) adjusted for age, sex, residence, education and presence of one or more risk factors for HCC by multiple logistic regression are reported in Table 4.

**Table 4:** Odds ratios (ORs) for hepatocellular carcinoma (HCC) according to distribution tertiles of polychlorinated biphenyls (PCBs) in the controls.

Lipid adjusted PCB concentration in serum (ng/g lipid )	Cases	Controls	OR (95% CI)*
PCB 118:			
T1 (0–11)	22	38	1
T2 (12–21)	10	32	0.90 (0.26–3.15)
T3 (22–241)	30	32	2.45 (0.85–7.07)
PCB 138:			
T1 (14–67)	21	34	1
T2 (68–106)	13	34	1.44 (0.44–4.74)
T3 (107–511)	28	34	2.42 (0.79–7.38)
PCB 153:			
T1 (23–121)	21	34	1
T2 (130–196)	12	34	1.31 (0.39–4.36)
T3 (206–691)	29	34	2.40 (0.79–7.31)
PCB 156:			
T1 (0–15)	28	35	1
T2 (16–24)	7	35	0.29 (0.08–1.00)
T3 (25–74)	27	32	1.10 (0.37–3.23)
PCB170:			
T1 (0–46)	23	34	1
T2 (47–71)	15	34	0.78 (0.25–2.47)
T3 (72–209)	24	34	1.61 (0.56–4.64)
PCB 180:			
T1 (34–163)	21	34	1
T2 (166–257)	15	35	1.05 (0.32–3.48)
T3 (261–810)	26	33	1.69 (0.57–4.99)
PCB 194:			
T1 (0–37)	16	35	1
T2 (39–63)	19	34	1.81 (0.55–5.99)
T3 (65–231)	27	33	4.75 (1.40–16.04)
Total PCBs:			
T1 (92–556)	24	34	1
T2 (569–908)	10	34	0.61 (0.18–2.07)
T3 (912–3089)	28	34	1.76 (0.62–5.03)

*Adjusted for age, sex, residence, education and presence of one or more risk factors for HCC by logistic regression analysis.

No statistically significant difference of the ORs was found for 3rd compared to 1st tertile of PCB distribution for all PCB congeners (p > 0.1), and for each congener except PCB 194 (p = 0.02). ORs higher than 1.5 or higher than 2 for 3rd compared to 1st tertile were found for almost all PCB congeners, although they were not statistically significant. Accordingly, no statistically significant linear trend of OR increase with increasing PCB serum levels was found for total PCBs and for all PCB congeners except PCB 194.

Similar results were found when restricting the analysis to subjects with, and subjects without, the main risk factors for liver disease (Table 5): increased ORs for 3rd vs 1st tertile of PCB distribution were observed for both groups, although the 95% CIs were large due to the small number of subjects in some categories.

**Table 5:** Odds ratios (ORs) for hepatocellular carcinoma (HCC) for total polychlorinated biphenyls by HCC risk factors, according to distribution tertiles of polychlorinated biphenyls (PCBs) in the controls.

Lipid adjusted PCB concentration in serum (ng/g lipid)	Cases	Controls	OR (95% CI)*
**No risk factor for HCC (HBV, HCV or 60 + g/day alcohol)**			
**Total PCBs**			
T1	4	31	1
T2	6	31	1.18 (0.27–5.12)
T3	9	30	1.60 (0.38–6.63)
**At least one risk factor for HCC (HBV, HCV or 60 + g/day alcohol)**			
**Total PCBs**			
T1	19	4	1
T2	4	3	0.19 (0.02–1.69)
T3	20	3	1.77 (0.29–10.56)

*Adjusted for age, sex and residence by logistic regression analysis.

We also performed subgroup analyses by gender, age and residence, which did not show substantially different results compared to the analysis of the whole dataset (data not shown in Table).

## Discussion

This preliminary study provides some evidence for an association between HCC and serum levels of PCBs, when also taking account of demographic and socio-economic factors and main risk factors for HCC, although the 95% confidence intervals of the estimates included the null value. These results are consistent for almost all the PCB congeners found in at least 30% of subjects. However, since HCC patients undergo liver cancer after a long history of cirrhosis, some concern may be raised whether the liver function impairment or the weight loss due to cancer development determine an increase of PCB serum levels in these patients^19^. In fact, we found that HCC cases who had lost more than 3 kg of body weight in the last 3 years, before HCC diagnosis, had higher PCB serum levels than those who had not lost weight, and we argued that higher PCB serum levels in the former were possibly due, at least partly, to mobilization of various chemicals, including PCBs, stored in body fat deposits. For this reason, we decided to exclude HCC cases with recent body loss of more than 3 kg.

Most HCC cases had one or more major risk factors for HCC, namely HCV and HBV infection and alcohol intake > 60 g/day for at least 10 years, in agreement with a previous case–control study on HCC aetiology carried out in the same area^13,20^. HCC cases with and without the main risk factors for the disease showed similar PCB serum levels, and the ORs for HCC did not vary substantially stratifying subjects according to the presence or absence of at least one risk factor for HCC, suggesting that PCBs may be independent risk factors for HCC development.

The association between HCC and PCB exposure is biologically plausible as the liver is a target organ of these chemicals and an increased incidence of liver cancer is observed after their administration in rats^2^. The different PCB congeners, including dioxin-like and non-dioxin-like compounds, have different activities according to their chemical structure. We found that moderate and high chlorinated PCB congeners were detectable in most subjects and were at the highest serum concentration, particularly congeners 138, 153, 170, 180 and 194, in agreement with those most commonly detected in human studies^2^.

However, PCBs usually occur in complex mixtures eliciting both genotoxic and non-genotoxic effects associated with carcinogenesis, tumour promotion and progression: in-vitro assays and experimental animal studies have shown that PCBs may produce oxidative stress and genotoxicity, interact with various receptors, including the aryl hydrocarbon receptor (AhR) and others controlling xenobiotic and steroid hormone metabolism or modulate plasma membrane-associated proteins affecting cell communication, adhesion and migration^2^.

In humans, an association between serum levels of PCB congeners and unexplained alanine aminotransferase (ALT) elevation was found in the US NHANES^5^, contrary to our findings of no relationship between PCB and ALT serum levels in the general population living in this area^21^. In the Anniston Survey, however, a cross-sectional study on a residential cohort with elevated PCB exposure, an association was found between serum levels of PCB congeners and those of the cytokeratin 18 biomarker, a more sensitive biomarker than ALT for environmental liver disease^4^.

Few epidemiological data are available on the possible association between PCB exposure and HCC at present. The cohort studies of people who ingested rice oil contaminated with PCBs and polychlorinated dibenzofurans (PCDFs) in the Yusho and Yucheng incidents, in Japan in 1968 and in Taiwan in 1979, found an excess mortality from liver cancer and liver cirrhosis among intoxicated subjects in the first 10–15 years after the poisoning outbreak^3,22^. However, almost 90% of the dioxin-like toxic equivalency (TEQ) in human blood of the contaminated people was contributed by PCDFs in both incidents^23^, arising doubts on the actual role of PCBs by themselves. On the contrary, although high levels of polychlorinated dibenzo-p-dioxins (PCDDs) and dibenzofurans (PCDFs) were also found in the general population in Brescia, the major contribution to TEQ serum levels was due to PCBs^8^. Some, but not all the occupational cohort studies found an increase in liver cancer mortality in PCB exposed workers with respect to national or regional figures^6,24^, but none of them had PCB serum measures.

The main strengths of our study are that it was performed in a highly industrialized area with both high incidence of HCC and heavy exposure to PCB and possibly PCDD and PCDF as a consequence of half a century of PCB diffusion in the environment, with particularly high concentrations of these chemicals in soil, food and blood of residents^7,8,10^.

This research has some limitations, too. Particularly, the limited number of HCC cases enrolled, due to the relatively high proportion of those excluded from the analysis for recent weight loss, which has reduced the study power of detecting an OR of at least 2 for 3rd compared to 1st tertile of PCB distribution. Therefore, our study can be considered as an explorative investigation on the association between HCC and PCB exposure.

The use of PCB serum level as a measure of the body burden of these chemicals in HCC patients may be of concern, due to their history of chronic liver disease. PCBs are highly soluble in lipids and are mainly deposited in adipose tissue and transported in the blood by lipids. HCC cases showed lower mean values of total cholesterol and triglycerides (133.5 and 85 mg/dl) than those found in the Italian population aged 65–74 years (204 and 132 mg/dl, respectively)^25^. This finding was expected, since low serum values of total cholesterol and triglycerides are usually found in HCC cases, due to advanced cirrhosis and diet followed before or during hospitalization for the liver disease^26^. In HCC patients, the partitioning between adipose tissue and serum is close to 1:1 only when both are expressed on a lipid basis. Indeed, in a previous study of 101 HCC incident cases enrolled in the same area, we found high correlations between serum and liver (Spearman r = 0.79), serum and fat (r = 0.91), and liver and fat (r = 0.75) concentrations of single PCB congeners and total PCBs, using lipid-adjusted PCB measures^27^. The retrospective study design allows the comparison only of present PCB serum levels between HCC cases and controls, ignoring past exposures to these chemicals. However, PCB serum levels are usually considered a valid measure of PCB body storage, as shown by the high correlations between PCB serum and adipose tissue levels found in our and other studies^27–31^.

As regards the selection of subjects, we enrolled both cases and controls by the same study base, i.e. residents in Brescia province, matched by age and gender, admitted to the same General Hospital. We collected blood samples of both groups at the same time and stored them for a short time before performing blinded laboratory analyses. The HCC cases were prospectively recruited among all the incident ones admitted to the General Surgery and Hepatology Divisions of the main hospital in the area, and they had similar characteristics as those enrolled in previous case–control studies on HCC etiology carried out in the same area^13,14^ and those reported by the Italian Cancer Registries^32^ and enrolled in a recent large multicentre Italian series^18^. We choose the controls among subjects hospitalized for traumatic causes, vascular surgery or minor surgery diseases. They showed similar PCB serum levels as the general population of the same age living in the town, as found in a recent survey^21^. Therefore, we are confident that no substantial selection bias exists in this study.

This preliminary study provides some, though not conclusive, evidence that PCB exposure may play a role in HCC development, independently of other risk factors.

## Methods

### Study population

We carried out a hospital-based case–control study. The study base was the population living in the province of Brescia and born in Italy. The study protocol established to enroll 100 HCC cases and 100 age- and gender-matched controls based on a-priori defined power of detecting an OR of at least 2 for 3rd compared to 1st tertile of PCB distribution at the threshold of p = 0.05 using two-tail statistical tests.

The cases were recruited consecutively and prospectively in two Divisions (General Surgery and Hepatology) of the main General Hospital of the province. 96% of the eligible cases agreed to participate in the study. Overall, we enrolled 102 patients with a first diagnosis of HCC (incident cases) in 2015–2018, before they underwent any treatment for the disease. The diagnosis of HCC was based on computerised tomography and was confirmed by histological analyses in 63 cases.

We selected 102 control subjects in the same age range and gender as the cases and without history of cancer or hepatic, endocrine or autoimmune diseases, who were admitted to the same hospital at the same time as the cases. When a subject suitable for the study according to the inclusion criteria was identified, he/she was invited to participate, and, in case of refusal, another individual was chosen and invited to participate. Overall, about 70% of the eligible subjects participated in the study. The enrolled controls were hospitalized for traumatic causes, vascular surgery or other minor surgery.

Both cases and controls provided a fasting blood sample for laboratory determination and were interviewed at the hospital on demographic variables, weight and height, residential and occupational history, smoking habit and alcohol intake by the research personnel. Since a rapid weight loss may determine an increase of PCB serum levels^33,34^, the HCC cases were also questioned about the quantity of, and time at, weight loss in the past.

Total alcohol intake was computed according to the average ethanol content of wine (12 percent by volume), beer (5 percent) and spirits (40 percent) and the frequency and quantity of alcoholic beverages consumed in the past. We considered the intake claimed by the subject during the decade in his/her lifetime with the highest consumption (“peak”), which had provided valuable results for evaluating the dose–effect relationship between alcohol intake and HCC occurrence in previous case–control studies carried out in this area^20^. Heavy alcohol intake was defined as consumption of 60 or more grams of ethanol per day for at least 10 years.

The Ethics Committee of the area approved the project (“Comitato Etico di Brescia”, ASST Spedali Civili, Brescia, Italy), and each participant provided a written informed consent. All research was performed in accordance with relevant guidelines/regulations.

### Laboratory analyses

The serum samples were stored at − 80° until and analyzed within 6 months since withdrawal. We investigated the following 33 PCB congeners, according to WHO classification^35^: 28, 31, 52, 74, 77, 81, 99, 101, 105, 114, 118, 123, 126, 128, 138, 146, 153, 156, 157, 167, 169, 170, 172, 177, 180, 183, 187, 189, 194, 196, 201, 203, 206 and 209.

The PCB analysis was conducted following a previously defined analytical method^36^, using an Agilent Technologies 6890 N gas chromatograph coupled with an Agilent Technologies MSD 5973 (electron impact ionization, mass filter: quadrupole). A PONA column (Agilent Technologies; 50 m × 0.20 mm ID) was used for chromatographic separation with carrier gas Helium. A 2 µL injection at 250 °C was performed by a 7683 Series Injector (Agilent Technologies) in splitless mode with a salinized injection liner (Agilent Technologies; 4 mm, 78.5 × 6.5 OD).

The limit of quantification (10 times the signal-to-noise ratio peaks) varied among PCBs but was generally less than 0.1 ng/ml for each congener.

PCB analysis was performed at the Laboratory of Occupational Hygiene and Toxicology, Brescia University, Italy. The Lab participated in inter-comparison programmes for toxicological analyses in biological materials (Institute for Occupational and Social medicine of the university of Erlangen-Nuremberg, D-91054 Erlangen, Germany), and fulfilled the requirements for congeners 28, 52, 101, 138, 153,180 in occupational and environmental medical fields T.

The instrument was calibrated using the standard solutions PCB-Mix 20 10 µg/mL in Isooctane and the PCB No. 30 10 µg/mL in Isooctane (both from Dr. Ehrenstorfer GmbH, Augsburg, Germany). As external quality control, the certified control serum 10 A/B by G-EQUAS (Friedrich-Alexander University, Erlangen, Germany) was included in each analysis.

Total PCB serum concentration was calculated as the sum of the 33 PCB congeners. Since PCB concentration is influenced by the amount of serum lipids, the ratio of PCB concentration to the total lipid levels was computed (lipid-adjusted PCB concentration) and expressed as ng/g lipid. We calculated the total lipid concentration from cholesterol and triglyceride levels using the formula proposed by Phillips et al.^37^: total serum lipid (g/L) = 2.27 * serum cholesterol (g/L) + triglycerides (g/L) + 0.623.

We also investigated the three main risk factors for HCC in this area, i.e. HBV and HCV infection and alcohol intake^10^, as potential risk modifiers of the possible association between PCB exposure and HCC. The presence of HBV and HCV infections was evaluated by testing sera for HBsAg and anti-HCV, respectively, using commercial immunoassays (EIA).

### Statistical analysis

The distributions of total PCBs and each congener serum levels were examined using common statistical techniques for exploratory analysis. Due to the asymmetric non-normal distribution of PCB values, the median, range and 90th percentile are reported. The differences in PCB concentration between HCC cases and controls were evaluated using non-parametric methods, particularly the Mann–Whitney test for impaired data. PCBs serum levels also were categorized into 3 categories, according to the tertiles of their distribution in the control group. Then the odds ratios (ORs) for HCC were computed for total PCBs and each congener as a measure of association between exposure and disease, as it usually done in case–control studies, assuming that the OR is a reasonable estimate of the relative risk. The reference category for computing the ORs was the lowest level (1st tertile). The ORs were calculated adjusting for sex, age, residence, education and presence of one or more risk factors for HCC, as they are often associated with PCB serum levels in the general population and therefore they are possible confounders of the association between PCB serum levels and HCC, using multiple logistic regression. The confidence intervals were computed at the 95% level. Furthermore, a test for linear trend was performed using the Wald test on the coefficients based on PCBs as continuous variables.

All the statistical tests were two-sided with a threshold of p = 0.05 for refusing the null hypothesis. The statistical analyses were performed using the STATA software for personal computer (Stata Statistical Software release 14.0; Stata Corporation, College Station, Texas).

## Supplementary Information

Supplementary Information

## Data Availability

Any data necessary to evaluation of the claims of the paper are provided as supplementary information.

## References

[1] ATSDR. Toxicological profile for polychlorinated biphenyls (PCBs). Agency for Toxic Substances and Disease Registry (ATSDR). U.S. Public Health Service, U.S. Department of Health and Human Services (2000). 36888731

[2] IARC. Polychlorinated Biphenyls and Polybrominated Biphenyls. Volume 107. IARC Monogr. Eval. Carcinog. risks to humans (2016). PMC7681612 29905442

[3] Tsai PC, Ko YC, Huang W, Liu HS, Guo YL (2007). Increased liver and lupus mortalities in 24-year follow-up of the Taiwanese people highly exposed to polychlorinated biphenyls and dibenzofurans. Sci. Total Environ..

[4] Clair HB (2018). Liver disease in a residential cohort with elevated polychlorinated biphenyl exposures. Toxicol. Sci..

[5] Cave M (2010). Polychlorinated biphenyls, lead, and mercury are associated with liver disease in American adults: NHANES 2003–2004. Environ. Health Perspect..

[6] Zani C, Toninelli G, Filisetti B, Donato F (2013). Polychlorinated biphenyls and cancer: An epidemiological assessment. J. Environ. Sci. Heal..

[7] Turrio-Baldassarri L (2007). A study on PCB, PCDD/PCDF industrial contamination in a mixed urban-agricultural area significantly affecting the food chain and the human exposure. Part I: Soil and feed. Chemosphere.

[8] Turrio-Baldassarri L (2008). PCDD/F and PCB in human serum of differently exposed population groups of an Italian city. Chemosphere.

[9] Turrio-Baldassarri L (2009). PCB, PCDD and PCDF contamination of food of animal origin as the effect of soil pollution and the cause of human exposure in Brescia. Chemosphere.

[10] Donato F (2006). Exposure to polychlorinated biphenyls in residents near a chemical factory in Italy: The food chain as main source of contamination. Chemosphere.

[11] Simonati C (2004). Cancer incidence and mortality in some health districts in Brescia area 1993–1995 [In Italian]. Ann. Ig..

[12] Forman, D. et al. Cancer incidence in five continents, vol. X. Scientific Publication. IARC Scientific Publications (2014).

[13] Donato F (1997). Hepatitis B and C virus infection, alcohol drinking, and hepatocellular carcinoma: A case-control study in Italy. Hepatology.

[14] Donato F, Gelatti U, Limina RM, Fattovich G (2006). Southern Europe as an example of interaction between various environmental factors: A systematic review of the epidemiologic evidence. Oncogene.

[15] Yang JD (2019). A global view of hepatocellular carcinoma: Trends, risk, prevention and management. Nat. Rev. Gastroenterol. Hepatol..

[16] Anstee QM, Reeves HL, Kotsiliti E, Govaere O, Heikenwalder M (2019). From NASH to HCC: Current concepts and future challenges. Nat. Rev. Gastroenterol. Hepatol..

[17] Younossi ZM (2019). Non-alcoholic fatty liver disease—A global public health perspective. J. Hepatol..

[18] Bucci L (2017). The evolutionary scenario of hepatocellular carcinoma in Italy: An update. Liver Int..

[19] Lim JS, Son HK, Park SK, Jacobs DR, Lee DH (2011). Inverse associations between long-term weight change and serum concentrations of persistent organic pollutants. Int. J. Obes. (Lond).

[20] Donato F (2002). Alcohol and hepatocellular carcinoma: The effect of lifetime intake and hepatitis virus infections in men and women. Am. J. Epidemiol..

[21] Agenzia di Tutela della Salute di Brescia (ATS Brescia). Esposizione a PCB nella popolazione dei comuni di Brescia, Castel Mella e Capriano del Colle. [In Italian]. Available at: https://legacy.ats-brescia.it/media/documenti/osservatorio_epidemiologico/21_DocumentiPubblicatiDaAllocare/PCB Sierici nel 2014 - Popolazione di Castel Mella e Capriano del Colle.pdf. (Accessed: 28th December 2019).

[22] Onozuka D, Yoshimura T, Kaneko S, Furue M (2009). Mortality after exposure to polychlorinated biphenyls and polychlorinated dibenzofurans: A 40-year follow-up study of Yusho patients. Am. J. Epidemiol..

[23] Masuda Y (1996). Approach to risk assessment of chlorinated dioxins from Yusho PCB poisoning. Chemosphere.

[24] Bosetti C, Negri E, Fattore E, La Vecchia C (2003). Occupational exposure to polychlorinated biphenyls and cancer risk. Eur. J. Cancer Prev..

[25] Istituto Superiore di Sanità. Il progetto cuore [In Italian]. Available at: http://www.cuore.iss.it/fattori/CuoreData.asp. (Accessed: 28th December 2019).

[26] Bircher J, Benhamou JP, McIntyre N, Rizzetto M (1999). Oxford Textbook of Clinical Hepatology.

[27] Zani C (2013). Polychlorinated biphenyls in serum, liver and adipose tissue of subjects with hepatocellular carcinoma living in a highly polluted area. Chemosphere.

[28] Stellman SD (1998). Relative abundance of organochlorine pesticides and polychlorinated biphenyls in adipose tissue and serum of women in Long Island New York. Cancer Epidemiol. Biomark. Prev..

[29] Covaci A (2008). Polybrominated diphenyl ethers (PBDEs) and polychlorinated biphenyls (PCBs) in human liver and adipose tissue samples from Belgium. Chemosphere.

[30] Bergonzi R (2009). Distribution of persistent organochlorine pollutants in maternal and foetal tissues: Data from an Italian polluted urban area. Chemosphere.

[31] Rusiecki JA (2005). A correlation study of organochlorine levels in serum, breast adipose tissue, and gluteal adipose tissue among breast cancer cases in India. Cancer Epidemiol. Biomarkers Prev..

[32] Associazione Italiana Registri Tumori (AIRTUM). I numeri del cancro in Italia 2018. [In Italian]. Available at: https://www.registri-tumori.it/cms/sites/default/files/pubblicazioni/2018_NumeriCancro-operatori.pdf. (Accessed: 28th December 2019).

[33] Chevrier J (2000). Body weight loss increases plasma and adipose tissue concentrations of potentially toxic pollutants in obese individuals. Int. J. Obes..

[34] Cheikh Rouhou M, Karelis AD, St-Pierre DH, Lamontagne L (2016). Adverse effects of weight loss: Are persistent organic pollutants a potential culprit?. Diabetes Metab..

[35] Van den Berg M (2006). The 2005 World Health Organization reevaluation of human and Mammalian toxic equivalency factors for dioxins and dioxin-like compounds. Toxicol. Sci..

[36] Turci R, Angeleri F, Minoia C (2002). A rapid screening method for routine congener-specific analysis of polychlorinated biphenyls in human serum by high-resolution gas chromatography with mass spectrometric detection. Rapid Commun. Mass Spectrom..

[37] Phillips DL (1989). Chlorinated hydrocarbon levels in human serum: Effects of fasting and feeding. Arch. Environ. Contam. Toxicol..

