# Supplementary material for: Polychlorinated biphenyls and risk of hepatocellular carcinoma in the population living in a highly polluted area in Italy
Source: Sci Rep. 2021 Feb 4;11:3064. doi: 10.1038/s41598-021-82657-8 (PMC7862237; doi:10.1038/s41598-021-82657-8)
Supplement: Supplementary file 1 — Supplementary Information [file 41598_2021_82657_MOESM1_ESM.docx]

Polychlorinated biphenyls and risk of hepatocellular carcinoma in the population living in a highly polluted area in Italy

Suppl. Table 1. Median, 90^th^ percentile and range of lipid-adjusted serum levels of polychlorinated biphenyl (PCB) congeners and total PCBs, in ng/g lipids, in unifocal and multifocal or diffuse HCC cases.

|  | **Unifocal HCC (n = 44)** | | | **Multifocal or diffuse HCC (n = 18)** | | | |
| --- | --- | --- | --- | --- | --- | --- | --- |
|  | **Median** | **90th percentile** | **Range (min-max)** | | **Median** | **90th percentile** | **Range (min-max)** |
| **PCB 74** | 0 | 24 | 0-37 | | 0 | 33 | 0-35 |
| **PCB 99** | 0 | 22 | 0-48 | | 0 | 44 | 0-49 |
| **PCB 118** | 19.5 | 60 | 0-83 | | 25 | 112 | 0-128 |
| **PCB 138** | 98.5 | 204 | 0-331 | | 85 | 347 | 0-349 |
| **PCB 146** | 8.5 | 24 | 0-35 | | 0 | 29 | 0-30 |
| **PCB 153** | 182 | 404 | 35-545 | | 142 | 622 | 39-699 |
| **PCB 156** | 22.5 | 42 | 0-63 | | 15 | 50 | 0-50 |
| **PCB 157** | 0 | 0 | 0-17 | | 0 | 14 | 0-15 |
| **PCB 167** | 0 | 11 | 0-16 | | 0 | 25 | 0-27 |
| **PCB 170** | 64.5 | 120 | 0-196 | | 52 | 163 | 0-176 |
| **PCB 172** | 0 | 13 | 0-26 | | 0 | 21 | 0-25 |
| **PCB 177** | 0 | 0 | 0-15 | | 0 | 0 | 0-0 |
| **PCB 180** | 236 | 476 | 52-779 | | 204 | 665 | 55-694 |
| **PCB 183** | 11 | 28 | 0-52 | | 0 | 46 | 0-57 |
| **PCB 187** | 31 | 64 | 0-123 | | 27 | 68 | 0-127 |
| **PCB 194** | 59 | 113 | 0-266 | | 51.5 | 142 | 0-180 |
| **PCB 196 + 203** | 31.5 | 60 | 0-108 | | 23 | 82 | 0-102 |
| **PCB 201** | 28 | 54 | 0-133 | | 22.5 | 53 | 0-80 |
| **PCB 209** | 0 | 19 | 0-53 | | 0 | 31 | 0-40 |
| **Total PCBs totali** | 834.5 | 1570 | 133-2613 | | 649.5 | 2596 | 95-2703 |
